# Intraoperative loading of calcium phosphate-coated implants with gentamicin prevents experimental *Staphylococcus aureus* infection *in vivo*

**DOI:** 10.1371/journal.pone.0210402

**Published:** 2019-02-01

**Authors:** Keith Thompson, Stoyan Petkov, Stephan Zeiter, Christoph M. Sprecher, R. Geoff Richards, T. Fintan Moriarty, Henk Eijer

**Affiliations:** 1 AO Research Institute Davos, Davos, Switzerland; 2 Spital Emmental, Burgdorf, Switzerland; Thomas Jefferson University, UNITED STATES

## Abstract

Orthopedic device-related infection (ODRI) is a potentially devastating complication arising from the colonization of the device with bacteria, such as *Staphylococcus aureus*. The aim of this study was to determine if intraoperative loading of a clinically approved calcium phosphate (CaP) coating with gentamicin can protect from ODRI *in vivo*. First, CaP-coated titanium aluminium niobium (TAN) discs were used to investigate the adsorption and release kinetics of gentamicin *in vitro*. Gentamicin loading and subsequent release from the coating were both rapid, with maximum loading occurring following one second of immersion, and >95% gentamicin released within 15 min in aqueous solution, respectively. Second, efficacy of the gentamicin-loaded CaP coating for preventing ODRI *in vivo* was investigated using a CaP-coated unicortical TAN screw implanted into the proximal tibia of skeletally mature female Wistar rats, following inoculation of the implant site with *S*. *aureus*. Gentamicin-loading prevented ODRI in 7/8 animals, whereas 9/9 of the non-gentamicin treated animals were infected after 7 days. In conclusion, gentamicin can be rapidly and simply loaded onto, and released from, CaP-based implant coatings, and this is an effective strategy for preventing peri-operative *S*. *aureus*-induced ODRI *in vivo*.

## Introduction

Despite advances in joint replacement and fracture fixation methodologies, orthopedic device-related infections (ODRIs) remain a major clinical concern. During joint replacement or fracture fixation procedures, commensal bacteria, such as *Staphylococcus aureus* or *S*. *epidermidis*, may enter the surgical site, potentially resulting in peri-operative infections (for review see [1]). Upon encountering the implanted material, the bacteria can rapidly adhere to the surface of the implant and form a biofilm, which renders the bacteria resistant to antibiotic therapy [2, 3].

In an attempt to prevent such infections from becoming established, antibiotics are frequently administered prophylactically, both systemically and locally [4]. For example, systemic prophylaxis, such as cefazolin administration during hip replacement surgery, reduces infection rates from 3.3% to 0.9% [5]. Despite this decrease in infection rate with systemic antibiotic administration, further reductions may be achieved via the local application of antibiotics. For example, antibiotic-loaded bone cement, polymethyl methacrylate (PMMA) [6], is successfully used to further decrease infection rates following total hip arthroplasty [7]. However, particular disadvantages of this approach include incomplete antibiotic release by PMMA cements, the necessity of surgical removal due to the non-biodegradable nature of PMMA [8], and the potential generation of antibiotic-resistant bacteria due to the prolonged nature of low-level antibiotic release from PMMA [9].

More recent strategies aimed at protecting the implant surface from colonization have also been devised, for example, hydrogels [10, 11], biodegradable coatings based on poly(D,L-lactic acid) [12, 13] or polypeptide nanofilms [14], and calcium-based bone substitutes [15] have all been investigated as antibiotic delivery vehicles. However, despite promising *in vitro* and *in vivo* findings with these approaches, only limited products have so far made it to the clinic, including gentamicin-loaded PDLLA-coated intramedullary nails, and an antibacterial hyaluronic acid-based hydrogel [16]. Furthermore, future development of new products is also hampered by the complex regulatory framework and cost barriers involved. The relatively simplistic approach of intraoperative admixing of antibiotic with bone cement is routinely performed, although specifically under the control of the surgeon. Therefore, there is a precedent for this approach which could be adapted for other implant coatings.

Coatings composed of calcium phosphate (CaP)/hydroxyapatite (HA) have been devised to enhance osseointegration of implanted dental or joint prostheses [17–19]. These coatings have no inherent antibacterial activity, but they exhibit a large surface area on which suitable antibacterial agents may adsorb, due to their crystalline nature and porosity. This raises the possibility that intra-operative loading of a CaP-based coating may provide a simple and effective means for targeted delivery of antibiotics, to protect the implant surface and thereby prevent peri-operative ODRI *in vivo*. The efficacy of such an approach has previously been demonstrated using intramedullary Kirschner wires (K wires) in a rabbit model [20], which demonstrated that a HA coating loaded with gentamicin (applied using inkjet technology) was effective at preventing *S*. *aureus* infection over 28 days. However, given the nature of a busy operating room, the use of a more simplistic approach, such as dipping the CaP-coated section of the implant in antibiotic solution immediately prior to implantation, would be more preferable than the need to employ more complex technologies such as inkjet spraying of coated implants.

Therefore, the aim of our study was to test whether a clinically used CaP-based implant coating could be effectively loaded with gentamicin intraoperatively by a simple dipping process and to determine the efficacy of such a strategy for preventing *S*. *aureus* ODRI *in vivo*.

## Materials & methods

### Materials

Clinically used gentamicin (Gentamicin 80, HEXAL AG, Holzkirchen, Germany) was provided as a 40 mg/ml solution. A clinical *Staphylococcus aureus* strain (JAR060131), isolated from a patient with an infected hip prosthesis [21], was used in the present study. The strain is broadly antibiotic susceptible, including gentamicin, except for resistance to penicillin (available at the Swiss Culture Collection, with accession number CCOS 890). Kirby-Bauer Zone of Inhibition assays were conducted using a gentamicin-sensitive *S*. *aureus* strain (NCTC 12973).

### Implant design and manufacturing

Custom-made discs (13 mm diameter, 2 mm thickness) and screws (4.4 mm in length; 1.4 mm in diameter) were machined from titanium aluminium niobium (TAN) alloy by RISystem AG (Davos, Switzerland). TAN disks and screws were coated with the BONIT coating (DOT GmbH, Rostock, Germany), a composite of brushite and HA (hereafter referred to as calcium phosphate: CaP), via electrochemical deposition. Before use, discs and screws were sterilized using a cold ethylene oxide cycle.

### Scanning electron microscopy (SEM) analysis

The surface morphology and the coating thickness of the CaP-coating was assessed by SEM using TAN discs and screws, respectively. The thickness of the coating was analyzed on a cross section of the screw, prepared following MMA embedding and cutting longitudinally through the midpoint of the screw. Prior to analysis, samples were prepared by mounting on a stub with a carbon sticker, followed by silver painting and coating with 10 nm carbon. Images were taken with a field emission SEM (S-4700, Hitachi, Tokyo, Japan) in secondary electron (SE) mode at an accelerating voltage of 3 kV, 40 μA emission current and a working distance of 12 mm. To identify the elemental composition of the materials, energy dispersive x-ray analysis (EDX) was performed at an accelerating voltage of 10 kV, 40 μA emission current and a working distance of 15 mm.

### Loading and release kinetics of gentamicin from the implant coating, and confirmation of antibacterial efficacy *in vitro*

Loading of gentamicin onto the implant coating was conducted by dipping coated TAN discs into gentamicin solution over a range of timepoints from 1 s to 60 min. Release of gentamicin into aqueous solution was then determined by placing the gentamicin-loaded discs into sterile PBS for an initial period of 15 min (0–15 min), followed by transfer into fresh PBS for a further 30 mins (15–45 min), then a further transfer into PBS for another period of 45 min (45–90 min), and finally a further 60 min period (90–150 min). Elution of the loaded gentamicin from the CaP coating was then quantified by spectrophotometry following derivatization with o-phthaldialdehyde, as previously described [11].

To confirm the eluted gentamicin retained antibacterial efficacy, and to support the spectrophotometric data, the elution solutions were further assessed using the Kirby-Bauer Zone of Inhibition (ZOI) Disk Diffusion Method [22] (based on EUCAST protocols for Antimicrobial Susceptibility Testing, using a methicillin- and gentamicin-sensitive *S*. *aureus* strain: NCTC 12973). Briefly, ZOI assays were conducted using tryptic soy agar (TSA: Oxoid, UK) plates coated with the control *S*. *aureus* strain (NCTC 12973), then a 20 μl aliquot of the respective aqueous solution was placed onto a blank filter disc (Sensi-Disc, BD Biosciences). Sensi-Discs containing 10 μg gentamicin were used as a positive control. The degree of growth inhibition was determined by measuring the diameter of the clear zone from the disc to the bacterial growth.

### Bacterial inoculum preparation for *in vivo* study

Bacterial stock cultures were stored at -80°C in 20% (v/v) glycerol. *S*. *aureus* (JAR060131 strain) was recovered from frozen stocks and cultured on TSA (Oxoid) or in tryptic soy broth (TSB: Oxoid) in ambient air at 37°C. The bacterial inocula were individually prepared in Phosphate Buffered Saline solution (PBS, Sigma-Aldrich, Switzerland) for each surgery, as previously described [23]. The *S*. *aureus* suspension was then adjusted to 5 x 10^8^ CFU/ml, thereby allowing an inoculum volume of 2 μl (corresponding to a target inoculum of 1 x 10^6^ CFU; acceptable range: 0.9 x 10^5^ to 1.5 x 10^6^) to be administered into the screw hole during the surgical implantation of the screw. All bacterial suspensions were prepared and stored at room temperature before transfer to the operating room and were used within a 4 h period. Quantitative culture of each inoculum was performed immediately after preparation to check the average CFU count.

### Animal welfare, observation and euthanasia

The study was approved by the ethical committee of the canton of Grisons in Switzerland (Approval numbers: 31_2015 & 16F_2016) and was carried out in an AAALAC-accredited research institute. Adult female, specific pathogen free (SPF) Wistar rats (17 weeks of age and mean animal weight at surgery = 306.9 g; range: 283.1–332.9 g), purchased from Charles River (Germany), were used in this study. A total of 24 animals were involved in the study. Animals were group housed in individually ventilated cages, with a 12h light/dark cycle and were fed *ad libitum* with 'mouse and rat maintenance' food (Kilba Nafag, Provimi Kliba AG, Extrudate 15mm round, #3436). Animal welfare was assessed using a scoring system based on a variety of aspects (general behavior, physical appearance, weight bearing on the operated limb, weight loss and wound healing), by scoring twice per day for the first three days, then once daily for the remainder of the study. After 7 days, animals were euthanized by intracardiac injection of pentobarbital under isoflurane anesthesia.

### Anesthesia, analgesia and surgery

Anesthesia and surgery were performed as described previously [24]. In brief, following peri-operative analgesia using buprenorphine (Temgesic) (0.1 mg/kg s.c.) and under isoflurane anesthesia, the rat was placed in dorsal recumbency and the skin over the left tibia was aseptically prepared. A 1 cm skin incision was then made on the proximolateral aspect of the left tibia. A unicortical hole was drilled 2 mm distal to the growth plate in the medial tibia using a 1.8 mm diameter drill bit (product #310.508, Depuy Synthes, Zuchwil, Switzerland). The drill hole was then tapped with a custom-made stainless-steel tap (2 mm outer/1.2 mm inner core diameter). At this point the bacterial inoculum was administered (2 μl volume containing 10^6^ CFU *S*. *aureus*) into the screw hole, for a total of 18 animals. For the 9 animals receiving the gentamicin-loaded screw, the CaP screw was placed into a 40 mg/ml gentamicin solution for 1 min before implantation. The remaining 9 animals received a CaP-coated screw without gentamicin loading. Following implantation into the screw hole, the screw head was then cut at the tip of the screw head. The fascia and the skin were closed in two layers using absorbable suture material (Monocryl and Vicryl rapid, Ethicon Inc., Cincinnati, USA; sizes 6–0 and 5–0, respectively). Post-surgery, animals received analgesia in the form of two further buprenorphine injections at 12h and 24h, in addition to paracetamol (Dafalgan Kindersirup, Bristol Myers Squibb) supplemented drinking water (2 mg/ml) for 5 days. Postoperatively, the animals were group housed under the same conditions, as previously described.

### Bacteriology

Following euthanasia 7 days after the surgery, the tibiae were dissected and the overlying fibrous tissue above the screw head, the screws and bones were collected in separate, sterile containers. The number of bacteria adhering to the screws was determined by sonicating the recovered screws for 3 minutes and vortex mixing for 10 seconds, before performing serial dilutions and viable bacteria counts on blood agar (Oxoid) plates. The entire tibia from each animal was then mechanically homogenized (Omni Tissue Homogenizer and Hard Tissue Homogenizing tips, Omni International, Georgia, USA) and the quantity of bacteria associated with bone was similarly quantified by serial dilution and performing viable bacteria counts on blood agar. Soft tissue samples were processed in the same manner. All agar plates were incubated for 24 hours at 37°C and all growth was checked for contamination or signs of co-infection. Animals were considered as infected when at least one sample (bone, soft tissue or screw) was culture-positive. Undiluted samples of tissue homogenates and sonicated implant fluid were also plated to confirm the culture-negative status of animals. Identification of *S*. *aureus* in culture-positive samples was confirmed using a Staphaurex^TM^ Latex Agglutination Test (ThermoFisher Scientific, UK).

### *In vivo* model of osseointegration

To address any potential inhibitory effects of gentamicin on osseointegration, a small pilot study (3 female Wistar rats per group (17 weeks old, mean animal weight at surgery = 296.8g; range = 270.8–333.2g; 2 groups) was conducted using CaP-coated screws, or CaP-coated screws dipped in gentamicin, implanted in the same model system in the absence of *S*. *aureus* infection, over an extended period of 28 days. Following euthanasia, the hind limb was removed and fixed in 70% ethanol for 2 weeks, dehydrated in an ascending series of ethanol washes, transferred into xylene and then embedded in MMA. Serial sections of ~270 μm thickness were then glued onto opaque Plexiglas slides, ground and polished (Exakt MicroGrinding System, Exakt Apparatebau, Germany) until a final thickness of ~140 μm was achieved. Slides were then stained with a 15% Giemsa solution (Fluka) followed by a 1% solution of Eosin (Sigma). Histomorphometric analysis on the blinded samples was performed by an independent assessor to quantify bone-implant contact (BIC) and bone density (bone volume/total volume: BV/TV) in a defined region 500 μm around the screw.

### Statistical analysis

Data is reported as mean ± S.D. unless stated otherwise. The Fischer Exact Test was used to check for differences in proportions of infected animals between groups. The Mann-Whitney test was used to analyze quantitative CFU data. Threshold for statistical significance was set as *p*<0.05. All calculations were performed using GraphPad Prism software (GraphPad Software, Inc., La Jolla, CA, USA).

## Results

### Morphological analysis of the CaP implant coating

**Fig 1:**
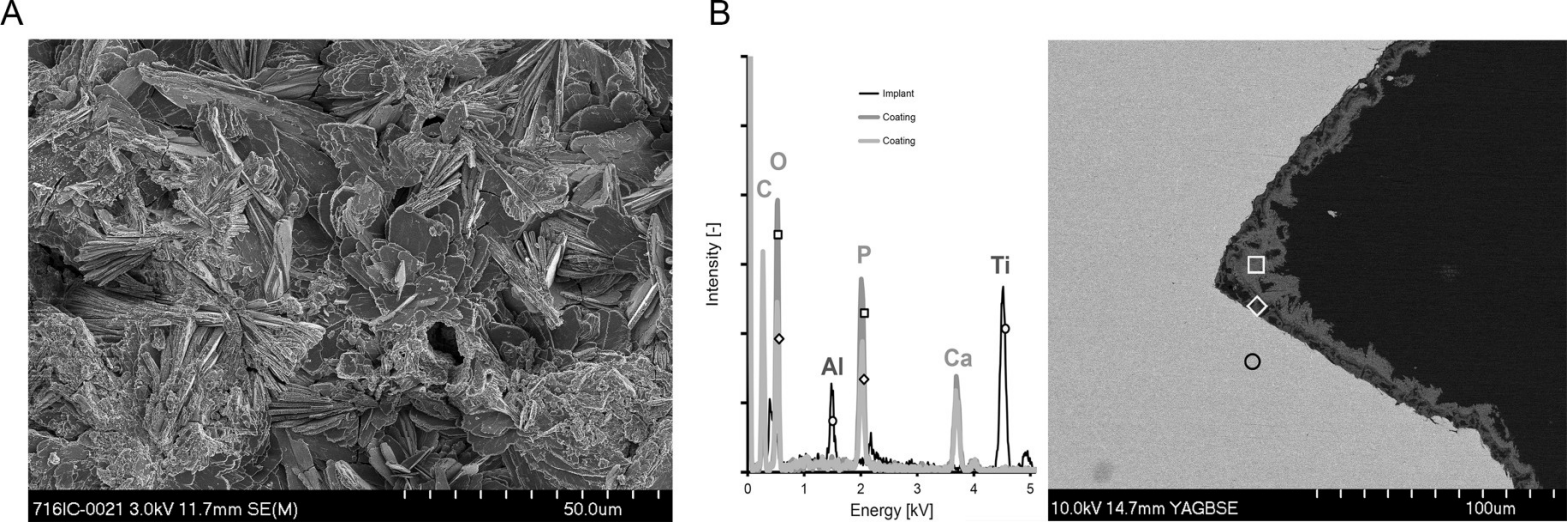
SEM analysis of surface morphology and coating thickness of the CaP coating used in the study. A.) Representative image of the CaP coating present on the TAN discs used during the *in vitro* aspect of the study. Scale bar is 50 μm. The image is from one disc and is representative of 2 further discs independently prepared and analyzed. B.) Left: Elemental composition of the CaP coating, as determined by energy dispersive X-ray analysis (EDX). The black line indicates the TAN screw, while the lower and upper layers of the coating are indicated by the light grey and dark grey lines, respectively. Right: Representative image of a longitudinal cross-section of the CaP-coated TAN screw (light grey) demonstrating the thickness of the CaP coating present on the screw and the regions of interest selected for the EDX analysis: screw–open black circle; lower level of CaP coating—open white diamond; upper layer of coating—open white square. Image is from one screw and is representative of 1 further screw, independently prepared and analyzed. Scale bar is 100 μm.

The structure of the CaP coating was investigated using SEM analysis. The electrochemical deposition method for application of the CaP-based coating resulted in a highly crystalline structure with large plate-like features, as evident on the CaP-coated TAN discs (Fig 1A), which is consistent with previous studies utilizing this particular CaP coating [19, 25]. Analysis of cross sections of CaP-coated screws revealed the coating thickness ranged from approximately 10 to 20 μm in depth (Fig 1B), as confirmed by EDX analysis.

### Gentamicin is rapidly adsorbed onto, and subsequently released from, the implant coating

**Fig 2:**
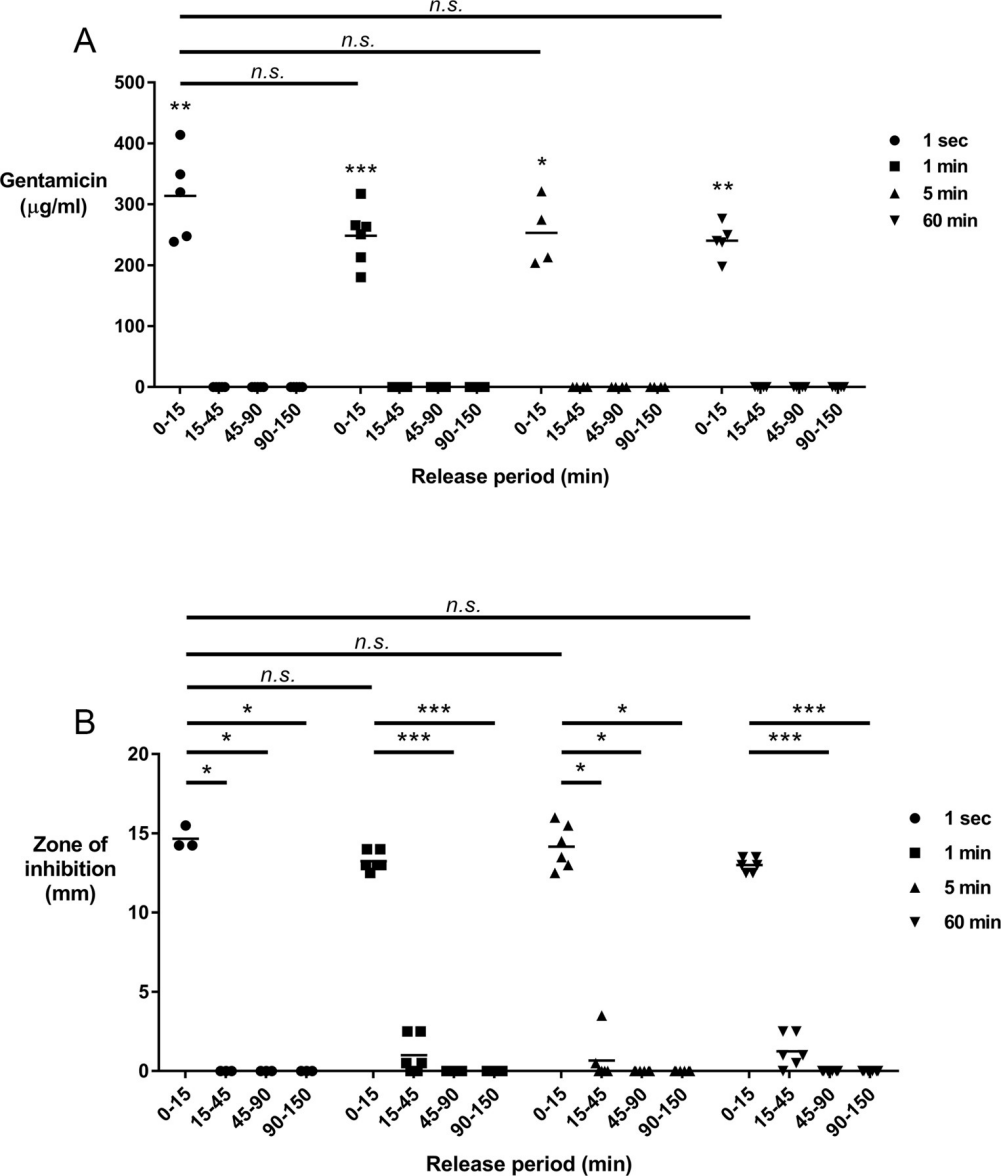
Characterization of gentamicin uptake, release, and antibacterial effects following loading onto the CaP coating. A.) Uptake and release kinetics of gentamicin loading of CaP-coated TAN discs. 13 mm diameter TAN discs coated with CaP were immersed in 40 mg/ml gentamicin solution for varying periods (See figure legend: 1 s–filled circles, 1 min–filled squares, 5 min–filled triangles, 60 min–filled inverted triangles) before transfer into sterile PBS for 15 min (0–15), followed by further serial transfers into fresh PBS for defined periods (15–45 min; 45–90 min; 90–150 min). The horizontal line indicates the mean from 4–6 individual experiments. B.) Antibacterial efficacy of the elution solutions from part A. Zone of inhibition (ZOI) assays involving a gentamicin-sensitive *S*. *aureus* strain were performed to confirm the gentamicin released from the CaP coating retained antibacterial efficacy. Data shown are the mean of 3–6 independent experiments. *p<0.05; **p<0.01; ***p<0.001; *n*.*s*. = not significant.

The potential clinical impact of utilizing such CaP implant coatings as delivery vehicles for antibiotics is at least partly reliant on the speed with which the coating may be loaded with such agents. As such we determined the capacity of the CaP coating for adsorbing the routinely used antibiotic gentamicin over a range of timepoints, from 1 s up to 60 min. We determined that the CaP coating rapidly adsorbed gentamicin from a 40 mg/ml solution, with a 1 s period being as effective as either a 1 min, 5 min or 60 min period (Fig 2A). Furthermore, upon transfer to aqueous solution, the adsorbed gentamicin is rapidly released from the coating within 15 min, with no further release detected over incubation periods conducted up to a total duration of 150 min. This suggests that a relatively rapid dipping period (≤1 min) of such CaP-coated implants will result in a burst-style release of gentamicin upon implantation *in vivo*.

To confirm the eluted gentamicin retained antibacterial efficacy we conducted ZOI assays to assess the inhibitory effects on a gentamicin-sensitive strain of *S*. *aureus* utilized in clinical antibiotic susceptibility tests. Consistent with our quantification data, the initial 15 min elution solutions from all dipping periods demonstrated the greatest degree of *S*. *aureus* growth inhibition, with only limited/no effect of the subsequent phases of elution period (>15 min) (Fig 2B). Interestingly, the 1 s dipping period also demonstrated equivalent efficacy compared to dipping periods ≥1 min, further validating the rapid nature of the gentamicin loading of the CaP coating. Given our findings we then decided to investigate if this approach could prevent *S*. *aureus*-induced ODRI in an *in vivo* model system and chose a 1 min dipping period for our subsequent studies.

### Gentamicin loading of CaP-coated implants prevents *S*. *aureus* ODRI *in vivo*

**Fig 3:**
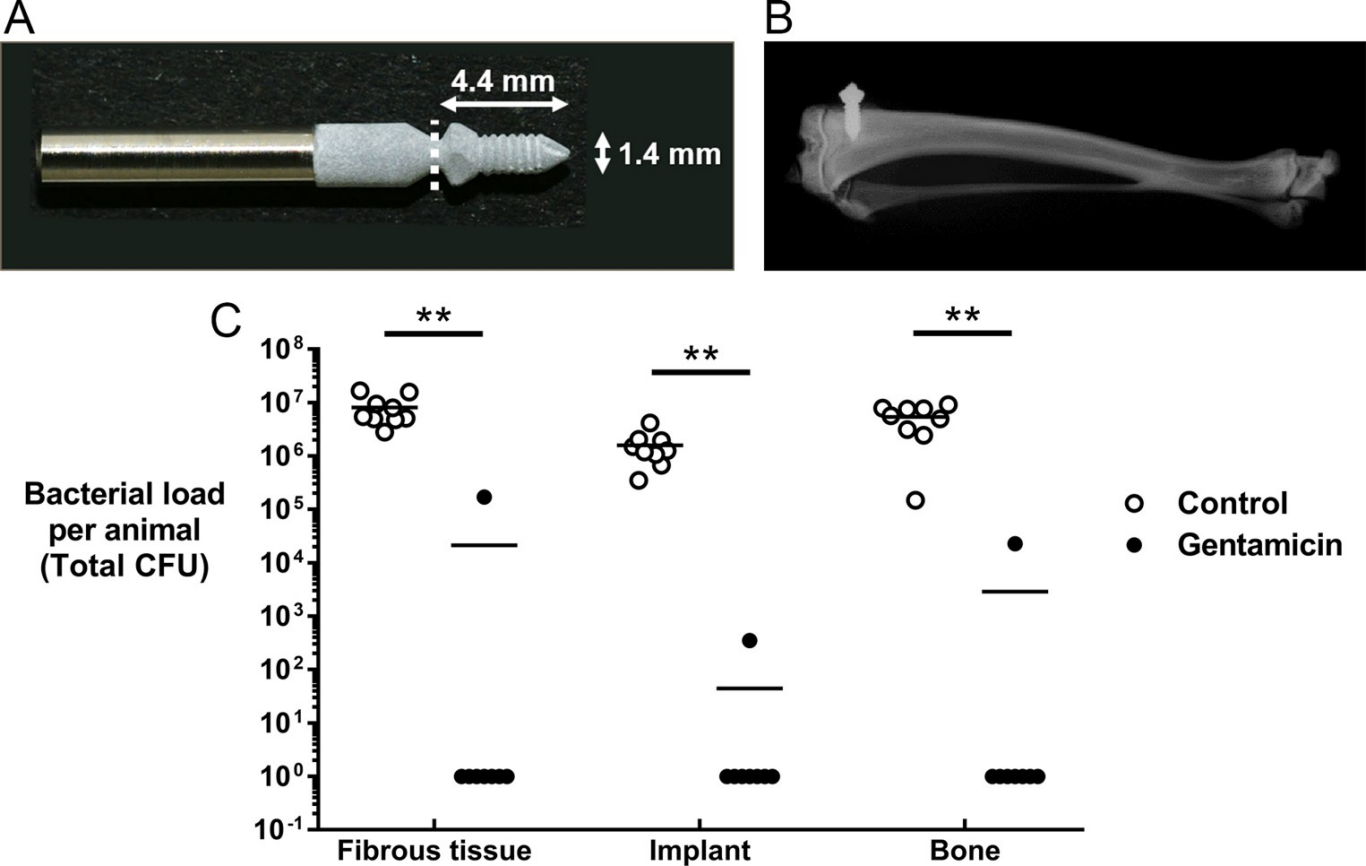
Efficacy of gentamicin-loaded CaP coating for preventing *S*. *aureus*-induced ODRI *in vivo*. A.) Representative images of CaP-coated TAN screws utilized in the study. White dashed line indicates the point at which the screws were cut following implantation *in vivo*. B.) Representative contact radiograph demonstrating the position of the implanted CaP-coated TAN screw (white) in the rat proximal tibia. C.) Quantitative bacteriology data from *S*. *aureus*-induced ODRI model. Skeletally mature female Wistar rats had a screw hole drilled into the proximal tibia which was inoculated with 10^6^ CFU *S*. *aureus* prior to implantation of a CaP-coated TAN screw with gentamicin loading (filled circles) or without gentamicin loading (open circles). After euthanasia at 7 days, the fibrous tissue overlying the screw (including any abscess; left panel), the implanted screw (middle panel), and the bone (right panel), were collected and processed for quantitative bacteriology. Culture-negative samples were assigned a CFU value of 1 to permit plotting on a log_10_ axis. **p<0.01.

We then used our previously characterized *in vivo* model of ODRI, utilizing a custom-designed screw (Fig 3A) implanted into the proximal tibia of a rat (Fig 3B) [24], to determine the efficacy of a 1 min dipping of CaP-coated TAN screws into gentamicin solution for preventing *S*. *aureus* infection.

After a 7-day incubation period, quantitative bacteriology revealed that all animals (9/9) receiving the screws without gentamicin dipping remained infected, with *S*. *aureus* detected in the soft tissue, bone, and were also found to have colonized the screw (Fig 3C). Total bacterial load of the non-gentamicin treated animals was consistently higher than the administered inoculum (mean CFU = 1.5 x 10^7^; range: 4.2 x 10^6^–2.6 x 10^7^). This was also associated with significant weight loss in the non-gentamicin treated animals of on average approximately 8% compared to an average weight loss of <1% in the gentamicin group (non-gentamicin group: mean weight at surgery = 306.0g ± 18.7; mean weight at euthanasia = 281.4g ± 21.0, **p = 0.0078; gentamicin group: mean weight at surgery = 310.8g ± 16.8; mean weight at euthanasia = 307.8g ± 13.3).

All infected animals in the non-gentamicin group demonstrated abscess formation, indicative of an ongoing infection. However, the majority of animals (7/8) receiving the gentamicin dipped screw were culture-negative for *S*. *aureus*, while the one infected animal had approximately two orders of magnitude lower levels of bacteria detected (total bacterial load = 1.9 x 10^5^ CFU), and lacked an observable abscess, compared to animals receiving the non-gentamicin dipped screws. One animal in the gentamicin group was excluded due to contamination with other bacterial species, possibly as a result of wound dehiscence during the experimental period in this animal. All bacteria recovered from the included animals was subsequently determined to be *S*. *aureus* using a Latex Agglutination Test kit. This demonstrates that the loading of CaP-coatings with gentamicin is an effective strategy for preventing peri-operative bone infections caused by gentamicin-sensitive *S*. *aureus* strains.

### Gentamicin loading does not inhibit osseointegration of CaP-coated implants in the absence of infection

**Fig 4:**
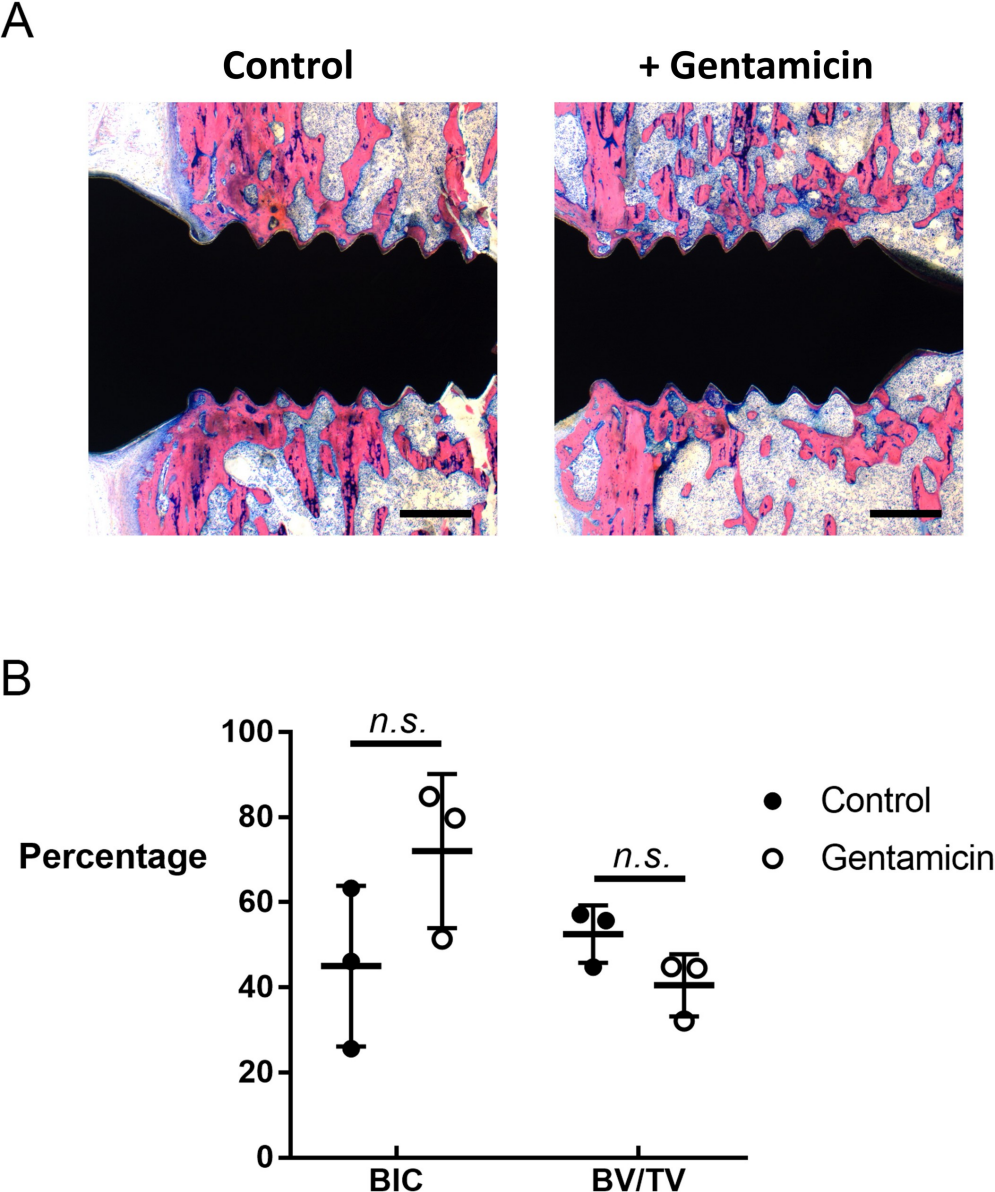
Effect of gentamicin loading on osseointegrative capacity of CaP coating in the absence of infection. CaP-coated TAN screws, with or without gentamicin loading, were implanted into the proximal tibia of skeletally mature female Wistar rats in the absence of infection for 28 days A.) Representative images of histological Giemsa-Eosin staining demonstrating BIC and BV/TV in the animals receiving CaP-coated TAN screws without gentamicin loading (left panel) or with gentamicin loading (right panel). Images are from one animal in each group and are representative of 2 further animals from each group. Scale bar = 500 μm. B.) Quantitative histomorphometric analysis of BIC and BV/TV in a defined region 500 μm around the screw. Data shown are from individual animals (mean ± S.D.; 3 animals per group).

To confirm that this strategy of gentamicin loading had no negative effect on the osseointegrative properties of CaP coatings we conducted a small pilot study (3 animals per group) utilizing the same *in vivo* model but in the absence of bacterial inoculation and over a longer (28-day) period. Histomorphometric analysis, using a defined region of interest around the screw, revealed that the presence of gentamicin on the CaP coating did not significantly reduce BIC, with mean BIC values of 72.1% (range: 51.4–84.9%) and 45.0% (range: 25.6–63.3%) observed for the presence or absence of gentamicin, respectively (Fig 4A and 4B). Similarly, bone density (BV/TV) in the proximity of the screw was also not significantly affected by the presence of gentamicin (gentamicin mean = 40.5%; range: 32.1–44.9%; *vs* no gentamicin mean = 52.5%; range: 44.8–57.1%). Therefore, despite the limited nature of this pilot study, we can suggest that the presence of gentamicin in the CaP coating does not have any significant inhibitory effects on bone formation or osseointegration in our experimental model up to a 28-day period.

## Discussion

Novel strategies aimed at protecting implanted devices from bacterial colonization are vital, given the increasing incidence of total joint replacement and fracture fixation surgeries necessary in increasingly aging populations. In this study, we have investigated the capacity of a CaP implant coating currently in routine clinical use to act as an antibiotic delivery vehicle for preventing *S*. *aureus*-induced ODRI. We determined that the CaP coating can be rapidly loaded with gentamicin via a simple dipping process, which is accompanied by a rapid burst-style release in aqueous solution. Furthermore, the gentamicin-loaded CaP coating dramatically protected from *S*. *aureus*-induced ODRI *in vivo*, without any demonstrable inhibitory effects of gentamicin on implant osseointegration, indicating the potential clinical translation of this approach.

Current clinical strategies for preventing peri-operative ODRIs fundamentally rely on both the local and systemic administration of prophylactic antibiotics [26]. However, the implant itself remains at risk of bacterial colonization for the duration of its lifetime *in vivo*. While the risk of hematogenous seeding of the implant by bacteria remains, in the case of closed fractures or total joint replacement surgeries, the greatest risk of bacterial colonization of the implant occurs during the surgical procedure itself. This suggests that effective short-term antibacterial approaches that are active at the implant surface may have dramatic effects at reducing the risk of developing a subsequent ODRI.

CaP coatings are attractive potential antibiotic delivery vehicles since they both promote osseointegration of the implant [19] and have been previously shown to be effectively loaded with gentamicin, which is subsequently released in a rapid burst-style *in vitro*, (although no *in vivo* anti-bacterial efficacy is reported in these studies) [27, 28]. While the prolonged release of gentamicin may actually be useful in the treatment of established osteomyelitis, as observed with an injectable CaP-based cement and gentamicin that effectively treated a rabbit model of osteomyelitis [29], a short duration or burst-style release may actually be of greater benefit for preventing peri-operative infections that normally develop in the critical days immediately after surgery. Indeed, such long-term retention, which is evident with some biodegradable bone cements and granules [30], as well as PMMA beads, may exacerbate the situation by promoting the development of antibiotic-resistant bacterial strains due to prolonged low-levels of antibiotic in the local microenvironment. Although other CaP-based composites including silica have been developed as bone fillers that report an initial burst-style release of gentamicin, this is accompanied by prolonged release periods of 28–70 days [27, 28]. Given our findings using the thin CaP implant coating (20 μm), and the fact that the particular CaP coating used in our study has previously been shown to be completely resorbed *in vivo* within 6 weeks [25], this suggests that long-term retention of gentamicin in the CaP implant coating does not occur, and that thin implant coatings are associated predominantly with a rapid burst release of gentamicin. This is indeed consistent with a previous study demonstrating that a thin (5 μm) gentamicin-loaded HA coating releases 95% of its gentamicin within 12h and 99% within 24h during *in vitro* elution testing [31].

Our results demonstrate that gentamicin may be effectively loaded into the CaP coating by simple dipping within a rapid time-frame (≤1 min), which is inherently compatible to the use of this approach in routine surgical procedures, without the need of any additional equipment. The observed lack of an inhibitory effect of gentamicin on osseointegration of the CaP-coated implant is consistent with previous studies reporting that gentamicin loading did not affect the function of osteoblasts cultured on brushite-coated titanium for producing bone matrix *in vitro* [32], and had no significant inhibitory effect on new bone formation or BIC in a rabbit model utilizing a HA-coated intramedullary K wire after 4 or 12 weeks [31]. Indeed, the lack of inhibitory effect is likely due to the rapid nature of the gentamicin release from the CaP coating, which, taken together, suggests they may be limited or no detrimental effects from using gentamicin in such clinical protocols.

A limitation could be levelled at our choice of the 7-day endpoint for the study, thereby raising the possibility that the infection may reoccur in apparently 'non-infected' animals. Due to a potential lack of sensitivity in our quantitative bacteriology assays, it is possible that some of our apparent 'non-infected' animals retain viable bacteria after gentamicin treatment, particularly given the recent reports of *S*. *aureus* being able to apparently colonize both murine [33] and human [34] sub-micron canaliculi. Consequently, should a more sensitive detection method have been employed, such as a PCR-based approach, this may reduce the apparent effectiveness of the gentamicin loading. Furthermore, given the aggressive nature of *S*. *aureus* as a pathogen *in vivo*, an extended duration study would require a much lower initial inoculum, to permit the non-gentamicin animals to withstand such a longer duration of infection. However, such a study design also requires far larger group sizes than those employed in this study, since a proportion of the animals receiving such lower inoculums will naturally clear the infection.

Currently we do not know the ability of other antibiotics to be loaded into the specific CaP coating under investigation, nor their *in vivo* efficacy for preventing ODRI. However, it has previously been demonstrated that the acidic or basic nature of the antibiotic can profoundly influence both its incorporation, and subsequent release from, a carbonated hydroxyapatite titanium coating [35]. Basic antibiotics, such as gentamicin and vancomycin, demonstrate reduced incorporation into, and faster release from, carbonated hydroxyapatite compared to acidic antibiotics, such as amoxicillin, carbenicillin and cephalothin. The authors hypothesized that this lack of incorporation and retention was potentially due to a lack of carboxyl groups in these basic antibiotics, resulting in reduced calcium binding affinity, which facilitated a burst-style release profile from the implant coating. Therefore, such CaP coatings may be capable of delivering a variety of antimicrobial agents and prove a useful tool in the fight against ODRI *in vivo*, although careful consideration should be given to the choice of agent and its potential issues with extended duration of release, such as the emergence of resistance and/or inhibitory effects on osseointegration.

## Conclusion

In conclusion, we demonstrate that a CaP coating, currently in widespread clinical use, can be rapidly loaded with gentamicin via a simple dipping process, which confers marked protection from *S*. *aureus*-induced ODRI *in vivo*. Such a strategy may provide a novel means for reducing the incidence of ODRI resulting from surgical procedures utilizing CaP-coated implants.

## Supporting information

S1 Table ARRIVE guideline checklist for in vivo studies. (DOCX)
