# Supplementary material for: Intraoperative loading of calcium phosphate-coated implants with gentamicin prevents experimental Staphylococcus aureus infection in vivo
Source: PLoS One. 2019 Feb 1;14(2):e0210402. doi: 10.1371/journal.pone.0210402 (PMC6358082; doi:10.1371/journal.pone.0210402)

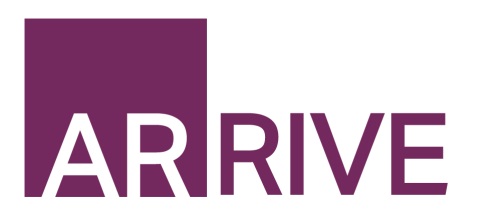


The ARRIVE Guidelines Checklist

Animal Research: Reporting In Vivo Experiments

Carol Kilkenny^1^, William J Browne^2^, Innes C Cuthill^3^, Michael Emerson^4^ and Douglas G Altman^5^

*^1^The National Centre for the Replacement, Refinement and Reduction of Animals in Research, London, UK, ^2^School of Veterinary Science, University of Bristol, Bristol, UK, ^3^School of Biological Sciences, University of Bristol, Bristol, UK, ^4^National Heart and Lung Institute, Imperial College London, UK, ^5^Centre for Statistics in Medicine, University of Oxford, Oxford, UK.*

|  | | ITEM | RECOMMENDATION | Section/ Paragraph |
| --- | --- | --- | --- | --- |
| 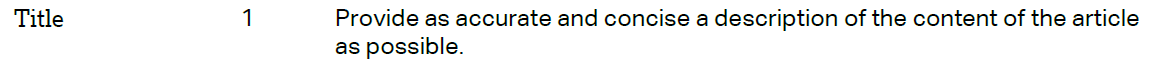 | | | ✓ |  |
| 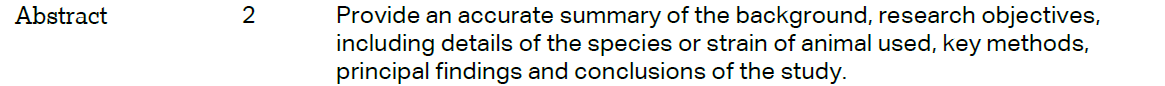 | | | ✓ |  |
| INTRODUCTION | | |  |  |
| 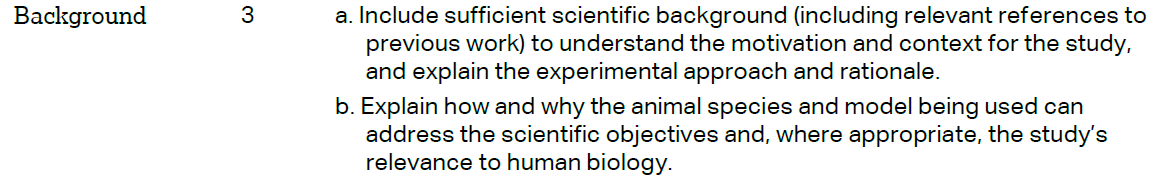 | | | Lines 71-78. |  |
| 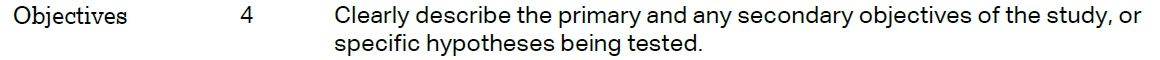 | | | Lines 93-95 |  |
| METHODS | | |  |  |
| 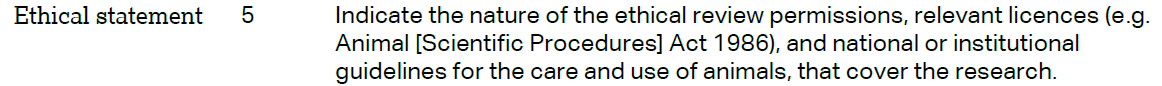 | | | Lines 163-165. |  |
| 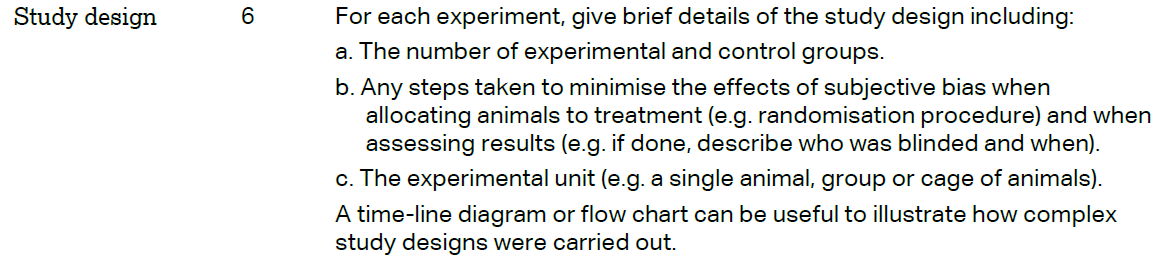 | | | Suppl Tables 1 & 2; Lines 180-184; 207-210  Lines 216-218. |  |
| 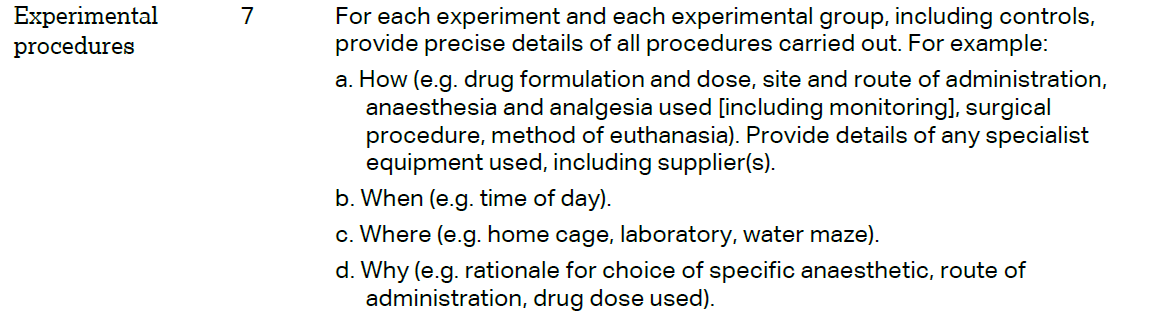 | | | Lines 174-189.  Not described (ND)  ND  ND |  |
| 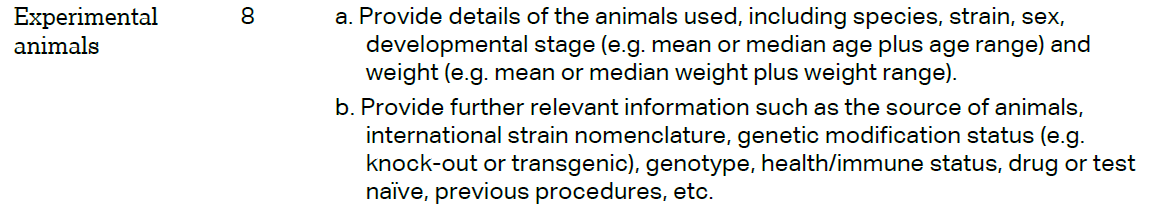 | | | Lines 165-168; 187-189.  Line 166. |  |

The ARRIVE guidelines. Originally published in *PLoS Biology*, June 2010^1^

| 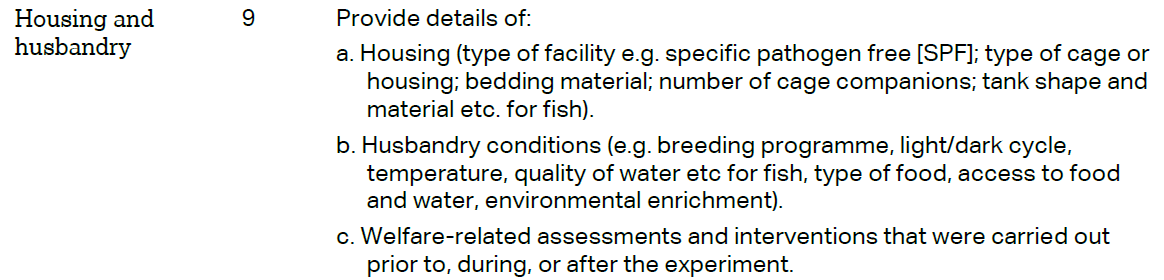 | Lines 164-165;  See Suppl text.  Lines168-171. | |
| --- | --- | --- |
| 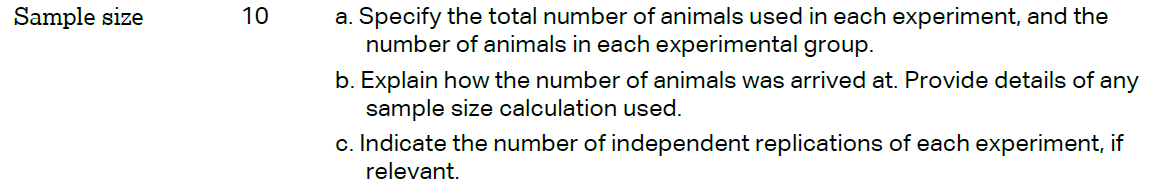 | See Suppl Tables 1 & 2; Lines 166-167.  See Suppl text. | |
| 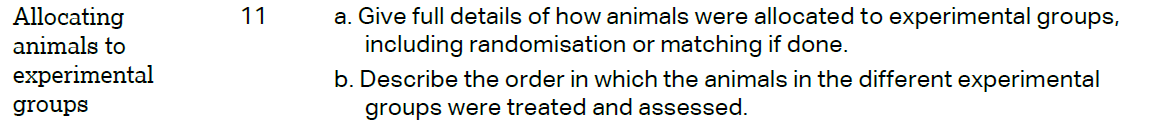 | N/A  N/A | |
| 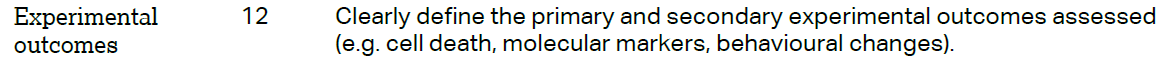 | Lines 201-203. | |
| 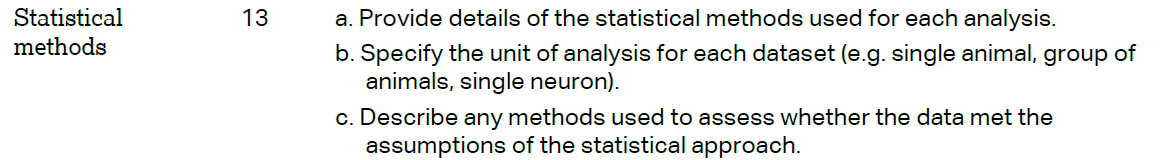 | In section 'Statistical analysis'. | |
| RESULTS |  | |
| 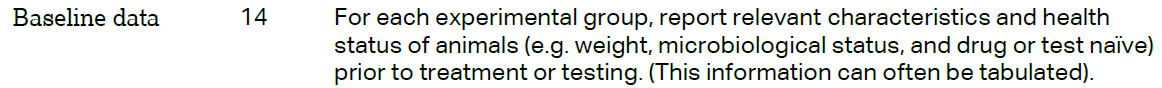 | Lines 188-189; Suppl Table 1 & 2. | |
| 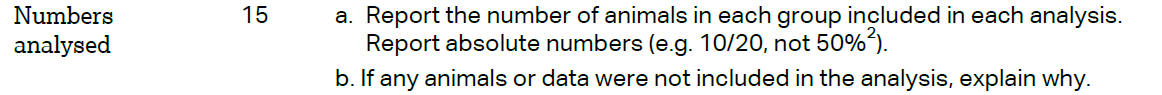 | Lines 272- 283. Figure legends (3 & 4).  Lines 286-288. | |
| 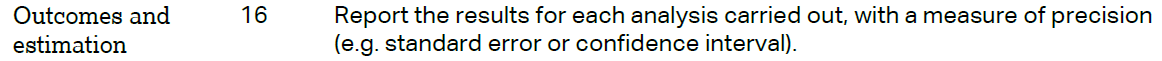 | Lines 276-286; 300-304. | |
| 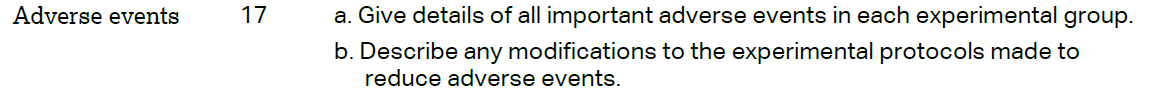 | N/A. | |
| DISCUSSION |  | |
| 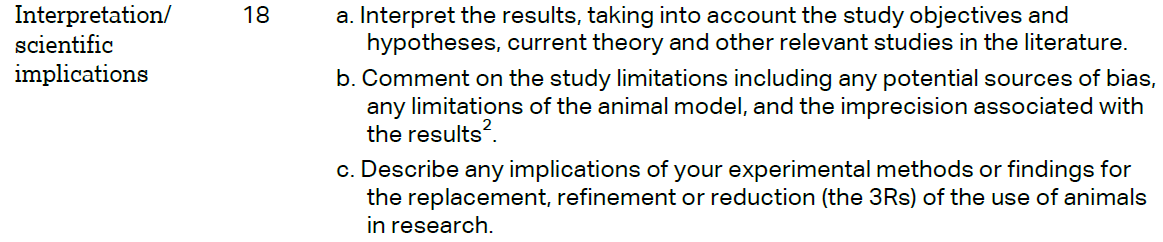 | ✓  Lines 359-373.  Lines 365-369. | |
| 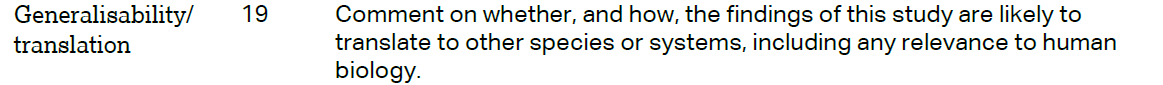 | Lines 386-390. | |
| 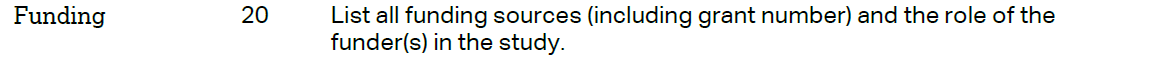 | | Lines 399-400. |


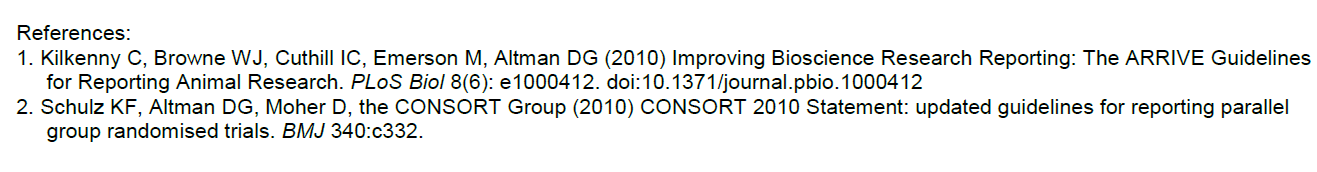

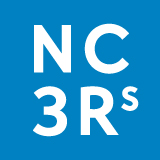

Supplement: S1 Table — (DOCX) [file pone.0210402.s001.docx]
